# Greater Mediterranean Diet Adherence Is Associated with Lower Odds of Chronic Migraine: A Retrospective Observational Study

**DOI:** 10.3390/nu18152563

**Published:** 2026-08-05

**Authors:** Ceren Alis, Ezgi Uludüz, Sevim Eyüpoğlu, Mustafa İskender, Onur Öztornacı, Aynur Özge, Derya Uludüz

**Affiliations:** 1 Department of Neurology, Sisli Hamidiye Etfal Training and Research Hospital, Istanbul 34371, Turkey; 2 School of Medicine, Koc University, Istanbul 33405, Turkey; euluduz22@koc.edu.tr; 3 Department of Psychology, Istanbul Atlas University, Istanbul 34403, Turkey; sevim.eyupoglu@atlas.edu.tr (S.E.);; 4 Independent Researcher, Kocaeli 41000, Turkey; ctfiskender@hotmail.com; 5 The MRC Integrative Epidemiology Unit, Department of Population Health Science, University of Bristol, Bristol BS8 1QU, UK; onur.oztornaci@bristol.ac.uk; 6 Nörom Neuroscience and Neurotechnology Center of Excellence, Ankara 06560, Turkey; 7 Department of Neurology, Faculty of Medicine, Istanbul Atlas University, Istanbul 34408, Turkey

**Keywords:** migraine, Mediterranean diet, water, sweetened carbonated beverages, coffee

## Abstract

**Background:** Dietary factors may influence migraine burden, but the relationship between Mediterranean diet (MD) adherence and migraine chronicity remains unclear. This study aimed to investigate whether higher MD adherence is associated with a less severe migraine phenotype, particularly lower odds of chronic migraine (CM) and fewer monthly headache days. **Methods:** This retrospective observational study included 448 patients diagnosed with migraine who were classified as having episodic migraine (EM) or CM. MD adherence was assessed using the validated Turkish version of the 14-item Mediterranean Diet Adherence Screener (MEDAS). Participants were categorized as having poor adherence (scores 0–5) or at least moderate adherence (scores 6–14). Demographic characteristics, migraine features, comorbidities, medication overuse, daily water intake, caffeine intake, sweetened carbonated beverage intake, and dietary behaviours were retrieved from patient files. Logistic regression models were used to evaluate the association between MD adherence and CM. Negative binomial regression was used for monthly headache days. **Results:** Of 448 patients, 256 had EM and 192 had CM. Patients with CM had lower MEDAS scores than those with EM and were less likely to have moderate-to-high MD adherence. Higher MD adherence was independently associated with lower odds of CM after adjustment for age, sex, BMI, disease duration, comorbidities, medication overuse, water intake, caffeine intake, and broader dietary behaviours (OR = 0.360, 95% CI: 0.193–0.674, *p* = 0.001). This association persisted after excluding patients with medication overuse, after multiple imputation, and when MEDAS was analysed continuously. Higher MD adherence was also associated with fewer monthly headache days in unadjusted and clinically adjusted models; however, this association was attenuated after full adjustment for caffeine intake and other dietary behaviours. **Conclusions:** Higher MD adherence was independently associated with lower odds of CM, even after adjustment for medication overuse and lifestyle-related dietary factors. The association with monthly headache days appeared more sensitive to broader dietary behaviours. These findings support the role of MD adherence as a potentially relevant lifestyle marker of migraine chronicity and warrant prospective studies that use detailed dietary assessment.

## 1. Introduction

Migraine is one of the most common neurological disorders, characterised by severe, throbbing, recurrent headaches and autonomic symptoms [1,2]. The aetiology of migraine involves a complex interplay of genetic and environmental factors, including weather conditions, stress, dietary choices, and dehydration [3,4,5].

The dietary dimension emerges as a potential influencer of the migraine phenotype, affecting attack frequency and severity by modulating systemic inflammation, vasodilation, and cerebral glucose metabolism [6,7,8]. Notably, certain foods such as wheat, citrus fruits, eggs, cheese, chocolate, coffee, red meat, alcohol, and sugar have been identified as common triggers for migraine attacks [9,10]. Elimination of these triggering foods has been shown to reduce migraine attack duration, frequency, and severity [9,10].

The Mediterranean diet emphasizes the consumption of plant-based foods such as vegetables, fruits, whole grains, legumes, nuts, and seeds, along with frequent intake of fish, seafood, and olive oil. It also allows for moderate consumption of poultry, eggs, dairy products, and alcohol, while limiting red meat intake [11]. While the Mediterranean diet has gained recognition for its preventive effects on various diseases, including cardiovascular disorders, diabetes, cancer, obesity, and Alzheimer’s disease [11], its impact on migraine remains an understudied domain [12,13]. In particular, the scarcity of investigations into the Mediterranean diet’s effects on migraine attack frequency, symptoms, and the transformation from episodic to chronic migraine necessitates further exploration.

Moreover, the relationship between caffeine, sweetened carbonated beverages, and migraine deserves thorough consideration. Caffeine, present in various beverages and foods, has been associated with both triggering and alleviating migraine symptoms, making its role complex and multifaceted [14,15]. Meanwhile, sweetened carbonated beverages, abundant in refined sugars and artificial additives, have been implicated as potential triggers for migraine attacks [16]. However, the intricate interplay between caffeine, sweetened carbonated beverages, and migraine requires in-depth exploration; scant literature currently exists on this subject.

Furthermore, despite the well-established association between dehydration and an elevated risk of migraine attacks, there exists a paucity of data regarding the direct influence of daily water consumption on the phenotypic expression of migraine [3,5]. Clarifying these relationships may improve understanding of the dietary and hydration factors associated with migraine phenotype and chronic migraine status. Accordingly, the primary objective of this study was to evaluate the association between Mediterranean diet adherence and chronic migraine status. Secondary objectives were to examine the association between Mediterranean diet adherence and monthly headache days and to explore the associations of daily water intake, caffeine intake, and sweetened carbonated beverage consumption with selected migraine-related clinical features.

## 2. Patients and Methods

### 2.1. Study Design and Population

We conducted a retrospective observational study using medical records of patients with migraine who presented to our clinic between October 2017 and May 2023. Migraine diagnosis and classification as episodic migraine or chronic migraine were established according to the International Classification of Headache Disorders, 3rd edition (ICHD-3) criteria [1]. Chronic migraine status was defined as the primary clinical outcome of the study.

The study was approved by the Ethics Committee of Sisli Hamidiye Etfal Training and Research Hospital (approval date and number: 16 January 2024–4235). The study was conducted in accordance with the ethical principles of the Declaration of Helsinki.

### 2.2. Data Collection

Demographic characteristics, migraine-related clinical features, migraine-associated symptoms, comorbid diseases, medication overuse, anthropometric measurements, dietary pattern, Mediterranean diet adherence, and daily intake of water, caffeine, and sweetened carbonated beverages were retrieved from patients’ files.

Demographic and anthropometric variables included age, sex, weight, height, and body mass index (BMI). BMI was calculated as weight in kilograms divided by height in meters squared when not directly available in the dataset.

Migraine-related variables included migraine type, age at migraine onset or diagnosis, disease duration, monthly headache days, attack duration without analgesics, pain intensity, and migraine-associated symptoms, including photophobia, phonophobia, nausea, vomiting, osmophobia, and allodynia. Pain intensity was assessed at the first visit using the Visual Analogue Scale (VAS). As part of the standard clinical protocol, all patients were asked to complete a 1-month headache diary after the initial visit to assess headache frequency before initiating any new treatment.

Medication overuse was recorded according to ICHD-3 criteria and defined as regular intake of acute headache medication on ≥10 days/month for triptans, ergotamines, opioids, or combination analgesics, or on ≥15 days/month for simple analgesics, for more than three months [1].

Comorbidities included hypertension, diabetes mellitus, depression, anxiety, and sleep apnea. Dietary and lifestyle variables included daily water intake, daily caffeine intake, daily sweetened carbonated beverage intake, skipping breakfast, eating snacks instead of proper meals, stress-related eating, irregular meal timing, and nocturnal eating.

### 2.3. Assessment of Mediterranean Diet Adherence and Dietary Behaviours

As part of the routine dietary assessment at the first clinical visit, patients completed the validated Turkish-language version of the 14-item Mediterranean Diet Adherence Screener (MEDAS) [17,18]. Each item was scored as 0 or 1 according to the published MEDAS scoring algorithm, and the unweighted item scores were summed to obtain a total score ranging from 0 to 14. Higher scores indicated greater adherence to the Mediterranean diet. The MEDAS total score documented in the clinical record was retrospectively extracted for the present analysis.

MEDAS scores were interpreted using the commonly applied categories of poor adherence (0–5), moderate adherence (6–9), and high adherence (≥10) [19,20]. For the primary analysis, participants were categorized as having poor adherence (MEDAS 0–5) or at least moderate adherence (MEDAS 6–14). This threshold was selected to distinguish poor adherence from at least moderate adherence while retaining adequate group sizes for multivariable analyses. To reduce dependence on a single categorical threshold, sensitivity analyses modeled MEDAS as a continuous score and additionally compared participants with high adherence (MEDAS ≥ 10) with those scoring <10. The MEDAS ≥10 analysis was considered exploratory because only 43 participants (9.6%) met the high-adherence criterion. Daily water intake was categorized as <1.5 L/day and ≥1.5 L/day. Daily caffeine intake was categorized as ≤300 mg/day and >300 mg/day. Daily sweetened carbonated beverage intake was categorized as daily consumer versus non-daily consumer.

At the first visit, patients completed five self-reported yes/no items included in the routine clinical dietary-habits form: “Do you skip breakfast?”, “Do you consume snacks instead of a main meal?”, “When you feel stressed, do you eat in an unhealthy manner?”, “Do you eat at irregular times?”, and “Do you eat at night?”. For each item, an affirmative response was coded as 1 and a negative response as 0. No frequency-based, quantity-based, or score-based cut-off was applied. Responses recorded as unknown or unavailable (code 99) or left blank were treated as missing. The five variables were entered separately as covariates and were not combined into a composite score. These items were routine clinical questions and were not derived from a validated dietary-behaviour scale.

### 2.4. Outcomes and Hypotheses

The primary outcome was chronic migraine status. The primary exposure of interest was adherence to the Mediterranean diet. The primary hypothesis was that higher adherence to the Mediterranean diet would be independently associated with lower odds of chronic migraine after adjustment for demographic factors, BMI, disease duration, comorbidities, medication overuse, water intake, caffeine intake, and other dietary-behaviour variables.

The secondary outcome was the number of monthly headache days. The secondary hypothesis was that higher adherence to the Mediterranean diet would be associated with fewer monthly headache days.

Additional exploratory hypotheses assessed whether daily water intake was associated with osmophobia, whether daily caffeine intake was associated with phonophobia or photophobia, and whether daily sweetened carbonated beverage intake was associated with migraine severity indicators, including chronic migraine, monthly headache days, pain intensity, and medication overuse.

### 2.5. Statistical Analysis

All statistical analyses were conducted using R version 4.3.2 (R Foundation for Statistical Computing, Vienna, Austria). Continuous variables were evaluated for distributional characteristics using visual inspection and the Shapiro–Wilk test. Descriptive statistics were presented as mean ± standard deviation or median with interquartile range for continuous variables, and as frequencies and percentages for categorical variables.

Baseline demographic, clinical, comorbidity, and dietary characteristics were compared between episodic migraine and chronic migraine groups. Continuous variables were compared using independent-samples *t*-tests or Mann–Whitney U tests, depending on distributional characteristics. Categorical variables were compared using chi-square tests or Fisher’s exact tests when expected cell counts were small.

Missing or non-applicable values were treated as missing. Listwise deletion was applied to regression models based on the variables included in each model. Because age, age at onset, age at diagnosis, and disease duration may be collinear, correlation structure and variance inflation factors were examined before multivariable modelling. Disease duration was retained in the main adjusted models when collinearity was acceptable.

For the primary outcome, chronic migraine status, binary logistic regression analyses were performed. The dependent variable was chronic migraine status, coded as episodic migraine = 0 and chronic migraine = 1. The main independent variable was Mediterranean diet adherence. Sequential models were constructed as follows:

Model 0: unadjusted model.

Model 1: adjusted for age, sex, and BMI.

Model 2: Model 1 plus disease duration.

Model 3: Model 2 plus depression, anxiety, hypertension, diabetes mellitus, and sleep apnea.

Model 4: Model 3 plus medication overuse.

Model 5: Model 4 plus daily water intake.

Model 6: Model 5 plus daily caffeine intake.

Model 7: Model 6 plus daily sweetened carbonated beverage intake, skipping breakfast, eating snacks instead of proper meals, stress-related eating, irregular meal timing, and nocturnal eating.

Odds ratios (ORs) with 95% confidence intervals (CIs) were reported. The persistence, attenuation, or loss of the Mediterranean diet association after the addition of medication overuse, water intake, caffeine intake, and broader dietary behaviours was specifically evaluated.

For monthly headache days, the distribution was examined for overdispersion. Because monthly headache days showed overdispersion, negative binomial regression was used. Chronic migraine status was not included as a covariate in these models because it is defined by headache frequency, and its inclusion would constitute overadjustment. Sequential models for monthly headache days followed the same adjustment strategy used in the chronic migraine models. Incidence rate ratios (IRRs) with 95% CIs were reported.

For each model, we reported the analytic sample size, the number of chronic migraine cases in the model-specific analytic sample, and the number of participants excluded from the original cohort because of missing model variables. Model fit was summarized using the Akaike information criterion (AIC) and Bayesian information criterion (BIC). Because analytic sample sizes differed across the sequential models, these indices were interpreted descriptively and were not directly compared across models with different sample sizes.

### 2.6. Missing-Data, Sensitivity, and Exploratory Analyses

Completeness of the variables included in Model 7 was evaluated, and participants included in the complete-case Model 7 analysis were compared with those excluded because of missing data. Continuous variables were compared using independent-samples *t*-tests or Mann–Whitney U tests, as appropriate, and categorical variables using chi-square or Fisher’s exact tests. Standardized differences were additionally calculated to describe between-group imbalance.

Given the substantial proportion of participants with incomplete Model 7 covariate data, multiple imputation by chained equations with predictive mean matching was performed as a missing-data sensitivity analysis. Fifty imputed datasets were generated under the missing-at-random assumption. The imputation model included chronic migraine status, monthly headache days, MEDAS score, and all covariates included in Model 7. Fully adjusted logistic and negative binomial regression models were fitted in each imputed dataset, and regression estimates and variances were pooled using Rubin’s rules.

To evaluate whether the findings depended on dichotomization of the MEDAS score, the total MEDAS score was entered into the sequential logistic and negative binomial regression models as a continuous variable. Effect estimates were reported per one-point increase in MEDAS score. Potential departure from linearity was explored by adding a quadratic MEDAS term and comparing the nested linear and quadratic models using likelihood-ratio tests. A second sensitivity analysis compared participants with high Mediterranean diet adherence, defined as MEDAS ≥10, with those scoring <10, using the same sequential logistic and negative binomial regression models.

To evaluate whether the association between Mediterranean diet adherence and migraine severity was driven by medication overuse, sensitivity analyses were performed after excluding patients with medication overuse. In this subgroup, logistic regression analyses for chronic migraine and negative binomial regression analyses for monthly headache days were repeated using the same adjustment framework, where model stability allowed.

For osmophobia, binary logistic regression was performed with osmophobia as the dependent variable and daily water intake as the main independent variable. The model was adjusted for age, sex, monthly headache days or migraine type, medication overuse, and Mediterranean diet adherence.

For phonophobia and photophobia, separate binary logistic regression models were performed with daily caffeine intake as the main independent variable. Models were adjusted for age, sex, monthly headache days or migraine type, medication overuse, and Mediterranean diet adherence.

Daily intake of sweetened carbonated beverages was evaluated as an exploratory exposure. Its associations with chronic migraine and medication overuse were assessed using logistic regression. Its association with monthly headache days was assessed using negative binomial regression, and its association with pain intensity was assessed using generalised linear modelling. These exploratory models were adjusted for clinically relevant covariates, including age, sex, BMI, Mediterranean diet adherence, and medication overuse, where appropriate. A two-sided *p*-value of <0.05 was considered statistically significant.

## 3. Results

### 3.1. Study Population and Baseline Clinical Characteristics

A total of 448 patients with migraine were included in the analysis. Of these, 256 patients were classified as having episodic migraine and 192 as having chronic migraine. The cohort consisted predominantly of women, with 373 female patients and 75 male patients.

Patients with chronic migraine were older than those with episodic migraine (44.0 [37.0–53.0] vs. 42.0 [35.0–49.0] years, *p* = 0.006). Sex distribution, BMI, and disease duration did not differ significantly between the episodic and chronic migraine groups. As expected, patients with chronic migraine had a substantially higher monthly headache frequency than those with episodic migraine (20.0 [15.0–30.0] vs. 8.0 [5.0–12.0] days/month, *p* < 0.001). Severe pain days per month were also higher in the chronic migraine group (15.0 [8.0–20.0] vs. 7.0 [4.0–10.0], *p* < 0.001).

Pain intensity measured by VAS did not differ significantly between groups. However, the attack duration without analgesics was longer in patients with chronic migraine (4.0 [4.0–24.0] vs. 4.0 [4.0–8.0] hours; *p* < 0.001). Medication overuse was markedly more frequent in chronic migraine than in episodic migraine (57.3% vs. 16.4%, *p* < 0.001). Migraine-associated symptoms, including photophobia, phonophobia, nausea, vomiting, osmophobia, and allodynia, did not differ significantly between groups, although allodynia showed a non-significant trend toward higher frequency in chronic migraine (8.9% vs. 4.7%, *p* = 0.076) (Table 1).

### 3.2. Comorbidities, Mediterranean Diet Adherence, and Dietary Behaviours

The frequency of hypertension, diabetes mellitus, depression, anxiety, and sleep apnea did not differ significantly between episodic and chronic migraine groups. Sleep apnea was numerically more frequent in chronic migraine, but this difference did not reach statistical significance (4.2% vs. 1.6%, *p* = 0.093).

Mediterranean diet adherence differed significantly according to migraine chronicity. Patients with chronic migraine had lower MEDAS scores than those with episodic migraine (4.0 [3.0–6.0] vs. 6.0 [4.0–8.0], *p* < 0.001). Moderate-to-high Mediterranean diet adherence, defined as MEDAS ≥6, was significantly less frequent in chronic migraine than in episodic migraine (28.1% vs. 51.2%, *p* < 0.001).

Daily water intake ≥1.5 L/day, daily caffeine intake >300 mg/day, and daily sweetened carbonated beverage intake did not differ significantly between groups. Among habitual dietary behaviours, skipping breakfast, stress-related eating, irregular meal timing, and nocturnal eating were not significantly different between episodic and chronic migraine. Eating snacks instead of proper meals was numerically more frequent in chronic migraine than in episodic migraine, showing a borderline association (23.1% vs. 15.6%, *p* = 0.056) (Table 2).

### 3.3. Mediterranean Diet Adherence and Chronic Migraine

In unadjusted logistic regression analysis, moderate-to-high adherence to the Mediterranean diet was associated with significantly lower odds of chronic migraine (OR = 0.373, 95% CI: 0.251–0.556, *p* < 0.001). This association remained significant after adjustment for age, sex, and BMI (Model 1: OR = 0.330, 95% CI: 0.216–0.507, *p* < 0.001), and after further adjustment for disease duration (Model 2: OR = 0.331, 95% CI: 0.216–0.508, *p* < 0.001).

The association was preserved after adding comorbidities, including depression, anxiety, hypertension, diabetes mellitus, and sleep apnea (Model 3: OR = 0.338, 95% CI: 0.220–0.520, *p* < 0.001). Importantly, the addition of medication overuse did not eliminate the association between Mediterranean diet adherence and chronic migraine (Model 4: OR = 0.346, 95% CI: 0.216–0.554, *p* < 0.001). The association also remained statistically significant after adjustment for daily water intake (Model 5: OR = 0.306, 95% CI: 0.188–0.499, *p* < 0.001), daily caffeine intake (Model 6: OR = 0.301, 95% CI: 0.172–0.526, *p* < 0.001), and broader dietary behaviors including sweetened carbonated beverage intake, breakfast skipping, snack-based meals, stress-related eating, irregular meal timing, and nocturnal eating (Model 7: OR = 0.360, 95% CI: 0.193–0.674, *p* = 0.001).

These findings indicate that higher Mediterranean diet adherence was independently associated with lower odds of chronic migraine, and that this association was not explained by demographic factors, BMI, disease duration, comorbidities, medication overuse, water intake, caffeine intake, or other dietary behaviours (Table 3).

### 3.4. Mediterranean Diet Adherence and Monthly Headache Days

Negative binomial regression was used to examine the association between adherence to the Mediterranean diet and the number of monthly headache days. In the unadjusted model, moderate-to-high Mediterranean diet adherence was associated with fewer monthly headache days (IRR = 0.814, 95% CI: 0.720–0.920, *p* = 0.001). This association remained significant after adjustment for age, sex, and BMI (Model 1: IRR = 0.777, 95% CI: 0.686–0.880, *p* < 0.001), and after additional adjustment for disease duration (Model 2: IRR = 0.778, 95% CI: 0.687–0.882, *p* < 0.001).

The association persisted after adjustment for comorbidities (Model 3: IRR = 0.788, 95% CI: 0.696–0.892, *p* < 0.001), medication overuse (Model 4: IRR = 0.825, 95% CI: 0.733–0.928, *p* = 0.001), daily water intake (Model 5: IRR = 0.817, 95% CI: 0.723–0.923, *p* = 0.001), and daily caffeine intake (Model 6: IRR = 0.855, 95% CI: 0.743–0.985, *p* = 0.030). However, after additional adjustment for sweetened carbonated beverage intake and habitual dietary behaviours, the association was attenuated and no longer statistically significant (Model 7: IRR = 0.883, 95% CI: 0.755–1.033, *p* = 0.120).

Thus, higher Mediterranean diet adherence was consistently associated with fewer monthly headache days in unadjusted and clinically adjusted models, but this association became non-significant after full adjustment for broader dietary behavior variables (Table 4).

### 3.5. Missing-Data and Multiple-Imputation Analyses

Of the 448 participants, 283 (63.2%) had complete data for all variables included in Model 7, while 165 (36.8%) were excluded from the complete-case analysis. Missingness was most frequent for daily caffeine intake (104/448, 23.2%), daily sweetened carbonated beverage intake (54/448, 12.1%), and the individual dietary-behaviour variables (10.3–11.2%).

Participants included in and excluded from Model 7 were compared using the available observations for each characteristic. Chronic migraine prevalence (43.1% vs. 42.4%, *p* = 0.888), MEDAS ≥6 prevalence (41.0% vs. 41.8%, *p* = 0.864), MEDAS total score (median 5 in both groups, *p* = 0.750), and monthly headache days (median 12 days in both groups, *p* = 0.877) were similar between the groups. Age, sex, BMI, and migraine disease duration also did not differ significantly. Among the observed covariates, depression (24.4% vs. 14.9%, *p* = 0.018; SMD = 0.240) and irregular meal timing (18.7% vs. 8.5%, *p* = 0.010; SMD = 0.303) were more frequent among participants included in Model 7. No statistically significant differences were observed for the remaining comorbidity and dietary-behaviour variables.

Given the extent of complete-case exclusion, multiple imputation using 50 imputed datasets was performed. In the multiply imputed fully adjusted logistic model, MEDAS ≥6 remained associated with lower odds of chronic migraine (OR = 0.293, 95% CI: 0.180–0.477, *p* < 0.001), consistent with the complete-case Model 7 estimate. For monthly headache days, the multiply imputed fully adjusted model showed an inverse association with MEDAS ≥ 6 (IRR = 0.803, 95% CI: 0.712–0.904, *p* < 0.001), whereas the corresponding complete-case estimate was not statistically significant. Thus, the chronic migraine finding was consistent across missing-data approaches, while the monthly headache-day finding was sensitive to the handling of missing data.

### 3.6. Sensitivity Analysis Excluding Patients with Medication Overuse

To determine whether the Mediterranean diet–migraine severity relationship was driven by medication overuse, sensitivity analyses were performed after excluding patients with medication overuse. In this subgroup, moderate-to-high Mediterranean diet adherence remained significantly associated with lower odds of chronic migraine in the minimally adjusted model (OR = 0.377, 95% CI: 0.212–0.669, *p* < 0.001), the clinically adjusted model (OR = 0.399, 95% CI: 0.222–0.715, *p* = 0.002), and the full lifestyle model (OR = 0.406, 95% CI: 0.186–0.889, *p* = 0.024).

For monthly headache days, moderate-to-high Mediterranean diet adherence remained associated with fewer headache days in the minimally adjusted model (IRR = 0.809, 95% CI: 0.687–0.954, *p* = 0.011) and the clinically adjusted model (IRR = 0.824, 95% CI: 0.699–0.970, *p* = 0.020). However, this association was attenuated and became non-significant in the full lifestyle model (IRR = 0.891, 95% CI: 0.723–1.098, *p* = 0.278).

These sensitivity analyses support the finding that the association between Mediterranean diet adherence and chronic migraine was not driven solely by medication overuse. In contrast, the association with monthly headache days appeared more sensitive to adjustment for broader lifestyle and dietary variables (Table 5).

### 3.7. Water Intake and Osmophobia

Daily water intake was evaluated in relation to osmophobia. Higher water intake, defined as ≥1.5 L/day, was not significantly associated with osmophobia in the model adjusted for monthly headache days (OR = 1.320, 95% CI: 0.884–1.972, *p* = 0.175). Similar results were obtained when chronic migraine status was used instead of monthly headache days in the model (OR = 1.301, 95% CI: 0.870–1.946, *p* = 0.201). Therefore, the hypothesis that lower daily water intake is associated with osmophobia was not supported in this dataset (Table 6).

### 3.8. Caffeine Intake, Phonophobia, and Photophobia

Daily caffeine intake >300 mg/day showed a borderline association with phonophobia. In the headache-days-adjusted model, higher caffeine intake was associated with higher odds of phonophobia, although this did not reach conventional statistical significance (OR = 1.733, 95% CI: 0.986–3.047, *p* = 0.056). A similar borderline result was observed in the chronic migraine-adjusted model (OR = 1.727, 95% CI: 0.983–3.036, *p* = 0.058).

In contrast, caffeine intake was not significantly associated with photophobia. The association was non-significant in both the headache-days-adjusted model (OR = 1.406, 95% CI: 0.664–2.978, *p* = 0.374) and the chronic migraine-adjusted model (OR = 1.394, 95% CI: 0.658–2.952, *p* = 0.385). These findings suggest a possible but statistically borderline relationship between higher caffeine intake and phonophobia, whereas no meaningful association was observed with photophobia (Table 6).

### 3.9. Sweetened Carbonated Beverage Intake and Migraine Severity Indicators

Daily intake of sweetened carbonated beverages was evaluated as an exploratory exposure. Daily consumption was not significantly associated with chronic migraine (OR = 0.903, 95% CI: 0.436–1.871, *p* = 0.784), monthly headache days (IRR = 1.087, 95% CI: 0.898–1.315, *p* = 0.390), pain intensity (β = −0.001, 95% CI: −0.305 to 0.302, *p* = 0.993), or medication overuse (OR = 0.874, 95% CI: 0.408–1.873, *p* = 0.730). Therefore, daily intake of sweetened carbonated beverages did not show a consistent or statistically significant relationship with migraine severity indicators in this cohort (Table 6).

### 3.10. Sensitivity Analysis Using MEDAS as a Continuous Variable

To evaluate whether the results depended on dichotomization of MEDAS scores, total MEDAS score was analyzed as a continuous variable. Each one-point higher MEDAS score was associated with lower odds of chronic migraine in the unadjusted model (OR = 0.890, 95% CI: 0.831–0.954, *p* < 0.001). The association remained significant throughout the sequentially adjusted models, including the fully adjusted model (Model 7: OR = 0.842, 95% CI: 0.758–0.936, *p* = 0.001). This estimate corresponds to approximately 15.8% lower odds of chronic migraine per one-point higher MEDAS score.

For monthly headache days, MEDAS score was not significantly associated with the outcome in the unadjusted model (IRR = 0.984, 95% CI: 0.963–1.005, *p* = 0.142) or in the fully adjusted model (Model 7: IRR = 0.989, 95% CI: 0.963–1.016, *p* = 0.433). Although an inverse association reached nominal significance in Model 5, it was not consistent across the sequential models. Adding a quadratic MEDAS term did not significantly improve model fit for chronic migraine or monthly headache days in either the unadjusted or fully adjusted models (all *p* > 0.20). These findings support the robustness of the association with chronic migraine without relying on an alternative categorical threshold, while the relationship with monthly headache days remained less consistent (Supplementary Table S1).

### 3.11. Sensitivity Analysis Using the Conventional High-Adherence Threshold

An additional sensitivity analysis was performed using the conventional high-adherence threshold of MEDAS ≥10. Overall, 43 of 448 participants (9.6%) met the high-adherence criterion, including 28 patients with episodic migraine and 15 with chronic migraine. In the fully adjusted Model 7, 28 participants with MEDAS ≥10 remained, of whom 11 had chronic migraine.

High adherence was not significantly associated with chronic migraine in the unadjusted model (OR = 0.690, 95% CI: 0.358–1.331, *p* = 0.269). The direction of the association remained inverse across the sequential models and became statistically significant after adjustment for medication overuse. In the fully adjusted model, MEDAS ≥10 was associated with lower odds of chronic migraine (Model 7: OR = 0.327, 95% CI: 0.119–0.894, *p* = 0.029).

High adherence was not associated with monthly headache days in either the unadjusted model (IRR = 1.008, 95% CI: 0.819–1.239, *p* = 0.943) or the fully adjusted model (Model 7: IRR = 0.974, 95% CI: 0.759–1.250, *p* = 0.836). Because the high-adherence group was relatively small and the confidence intervals were wide, these findings should be interpreted as exploratory.

## 4. Discussion

This retrospective observational study investigated the associations of Mediterranean diet adherence, daily water intake, daily caffeine consumption, and sweetened carbonated beverage intake with migraine phenotype and chronic migraine status. Chronic migraine status was used as the primary clinical marker of progression to chronicity, while monthly headache days were evaluated as a secondary severity outcome. The main finding was that higher Mediterranean diet adherence was independently associated with lower odds of chronic migraine. This association remained significant after sequential adjustment for demographic factors, BMI, disease duration, comorbidities, medication overuse, daily water intake, daily caffeine intake, sweetened carbonated beverage intake, and broader dietary behaviours. Importantly, the association with chronic migraine also persisted after excluding patients with medication overuse and when MEDAS was modelled as a continuous variable. In the fully adjusted continuous-score analysis, each one-point higher MEDAS score was associated with lower odds of chronic migraine, whereas no significant association was observed with monthly headache days. This pattern supports a more consistent relationship with chronic migraine status than with headache-day count and reduces concern that the main finding was dependent on the selected categorical threshold. In contrast, the association between Mediterranean diet adherence and monthly headache days was significant in unadjusted and clinically adjusted models, but was attenuated after full adjustment for caffeine intake and other dietary behaviours. Daily water intake was not associated with osmophobia, caffeine intake showed only a borderline association with phonophobia, and sweetened carbonated beverage intake was not associated with migraine severity indicators.

The inverse association between Mediterranean diet adherence and chronic migraine was also preserved after multiple imputation of missing Model 7 covariates. The pooled estimate was consistent with the complete-case fully adjusted estimate, reducing concern that the primary finding resulted from selective complete-case inclusion. In contrast, the association with monthly headache days differed according to the missing-data approach: it was not significant in the complete-case fully adjusted analysis but was significant after multiple imputation. Therefore, conclusions regarding monthly headache days remain less robust and should be interpreted cautiously.

The additional analysis using the conventional high-adherence threshold of MEDAS ≥10 provided a more cautious picture. High adherence was not significantly associated with chronic migraine in the unadjusted analysis, although the association was inverse and became statistically significant after adjustment for medication overuse and other covariates. No association was observed with monthly headache days. Importantly, only 43 participants met the MEDAS ≥10 criterion, and only 28 remained in the fully adjusted analysis. Therefore, the wide confidence intervals and limited number of highly adherent participants require cautious interpretation. These findings support retaining MEDAS ≥6 as the primary threshold for distinguishing poor adherence from at least moderate adherence, while considering the MEDAS ≥10 comparison as an exploratory sensitivity analysis.

The primary finding of this study was an inverse association between Mediterranean diet adherence and chronic migraine status. Patients with chronic migraine had lower MEDAS scores and were less likely to have at least moderate Mediterranean diet adherence than patients with episodic migraine. However, because dietary adherence and migraine status were assessed within a retrospective observational design, the temporal direction of this relationship cannot be established. The findings therefore do not demonstrate that greater Mediterranean diet adherence prevents migraine chronification. Reverse causation and residual confounding by unmeasured health, behavioural, or socioeconomic factors remain possible, and the observed association should be considered hypothesis-generating.

Our findings are consistent with the growing literature suggesting that overall diet quality and healthy dietary patterns are related to migraine burden. Previous studies have shown that lower adherence to Mediterranean or DASH dietary patterns is associated with more severe and frequent migraine attacks, higher disability, and greater headache impact [12,13]. A recent study has also suggested that poor adherence to the Mediterranean diet, particularly when accompanied by sleep disturbance and other lifestyle factors, may be associated with migraine chronification and disability [20]. Our study extends this literature by showing that Mediterranean diet adherence is not only related to headache burden but remains independently associated with chronic migraine status after adjustment for medication overuse and several dietary and lifestyle-related variables.

Previous literature has proposed inflammatory, oxidative, metabolic, and neurovascular pathways through which Mediterranean dietary patterns could potentially influence migraine [6,7,8,21]. However, the present study did not measure inflammatory or oxidative biomarkers, vascular function, mitochondrial activity, metabolic mediators, neuroinflammation, or CGRP-related pathways. Our data therefore cannot determine whether any of these proposed mechanisms explain the observed association. The relationship may also reflect correlated health behaviours, reverse causation, or residual confounding. Mechanistic explanations should consequently be regarded as hypotheses requiring evaluation in prospective studies incorporating relevant biological measurements. The persistence of the Mediterranean diet association after accounting for medication overuse in the model is particularly important. Medication overuse is one of the strongest modifiable factors associated with progression to chronic migraine. In our cohort, medication overuse was substantially more frequent in patients with chronic migraine, as expected. However, the association between Mediterranean diet adherence and chronic migraine remained significant after adjustment for medication overuse and after excluding patients with medication overuse. This suggests that the Mediterranean diet–chronic migraine relationship is not explained solely by excessive acute medication intake.

The association between Mediterranean diet adherence and monthly headache days was less consistent across the analytical approaches. In the complete-case fully adjusted model, the association was attenuated and was not statistically significant, whereas an inverse association was observed after multiple imputation. This difference indicates sensitivity to the handling of missing data. It should not be interpreted as evidence that monthly headache days are biologically less valid or that chronic migraine status is an inherently more stable or robust endpoint. Chronic migraine status and monthly headache days represent related but non-interchangeable clinical outcomes and were analyzed using different statistical models. Our findings support only a more consistent statistical association with chronic migraine status in the analyses performed here; they do not establish the comparative biological validity of the two outcomes.

Migraine dietary research may benefit from evaluating overall dietary patterns alongside individual foods and beverages. The predictive value of self-reported dietary triggers is variable and may differ substantially between individuals [14,15,16,22,23,24]. Pattern-level instruments such as MEDAS capture several dimensions of diet simultaneously, whereas individual intake variables represent narrower exposures. In the present study, MEDAS was associated with chronic migraine status, while water, caffeine, and sweetened carbonated beverage intake did not show consistent associations with the evaluated migraine characteristics. This descriptive comparison should not be interpreted as evidence that overall dietary patterns are biologically more important than individual dietary exposures. Water intake was evaluated because dehydration has long been considered a potential trigger or aggravating factor for headache and migraine [3,5,21]. Previous studies have suggested that increasing daily water intake may reduce headache intensity or headache hours, and observational data have linked higher water intake with lower headache frequency, lower disability, and shorter attack duration [3,5]. Based on this background, we hypothesized that lower daily water intake might be associated with osmophobia. However, this hypothesis was not supported in our cohort. Daily water intake of ≥1.5 L/day was not significantly associated with osmophobia after adjustment for age, sex, migraine severity, medication overuse, and Mediterranean diet adherence.

This negative finding should not be interpreted as evidence that hydration is irrelevant in migraine management. Rather, it suggests that osmophobia may not be a simple clinical marker of low water intake. Osmophobia is likely influenced by central sensory processing, attack phase, migraine subtype, and individual sensory susceptibility. Moreover, retrospective self-reported water intake may not accurately reflect hydration status, total fluid intake, electrolyte balance, climate exposure, sweating, or acute dehydration before attacks. Therefore, although adequate hydration remains a reasonable, low-risk lifestyle recommendation, our findings do not support a direct, independent association between daily water intake and osmophobia.

Caffeine showed a complex and symptom-specific pattern. Higher daily caffeine intake was not associated with photophobia and showed only a borderline association with phonophobia. This finding is consistent with the broader literature showing that caffeine has a dual role in headache disorders. Caffeine may have acute analgesic properties and is included in some headache medications, but high intake, irregular intake, or abrupt withdrawal may also provoke headache in susceptible individuals [14,15]. Population-based data have suggested modest and sometimes conflicting associations between caffeine consumption and headache prevalence or frequency [14]. Therefore, the borderline association between caffeine intake and phonophobia in our cohort should be interpreted cautiously and considered exploratory.

The absence of a significant association between caffeine and photophobia is also notable. Photophobia and phonophobia are both core migraine-associated symptoms, but they may not share identical clinical or behavioural correlates. Our findings do not support a generalized relationship between high caffeine intake and sensory hypersensitivity. Instead, if caffeine is related to migraine-associated sensory symptoms, the association may be subtle, heterogeneous, and influenced by individual susceptibility, withdrawal patterns, sleep behaviour, medication use, or headache phase. Future diary-based studies are needed to determine whether caffeine intake temporally precedes sensory symptoms or whether higher caffeine intake simply reflects coping behaviour among patients with a greater migraine burden. Daily sweetened carbonated beverage intake was not associated with chronic migraine, monthly headache days, pain intensity, or medication overuse. This finding suggests that daily consumption of sweetened carbonated beverages was not a major determinant of migraine phenotype or progression to chronicity in this cohort. Although some studies and reviews have discussed artificial sweeteners, additives, caffeine-containing beverages, and processed foods as potential headache triggers, the evidence remains inconsistent and often relies on self-reported perceptions of triggers [16,22,23,24]. The lack of a significant association in our data supports the interpretation that isolated beverage exposures may be less informative than overall dietary patterns. Therefore, intake of sweetened carbonated beverages should be considered an exploratory lifestyle variable rather than a central explanatory factor in this study.

These findings suggest that overall diet quality and related lifestyle behaviours may be considered as part of a comprehensive clinical assessment of patients with migraine. However, the observational design does not demonstrate that adopting a Mediterranean diet prevents or treats chronic migraine. Dietary counselling may continue to follow established general health and cardiometabolic recommendations, but migraine-specific recommendations based on the present findings would be premature. Prospective studies and structured dietary interventions are needed to determine whether improving Mediterranean diet adherence can modify migraine-related outcomes. This study has several limitations. First, its retrospective observational design precludes causal inference. Therefore, we cannot determine whether higher Mediterranean diet adherence reduces the risk of progression to chronic migraine or whether patients with less severe migraine are more likely to maintain healthier dietary patterns. Second, dietary variables were obtained from self-reported clinical questionnaires, which may be subject to recall bias and misclassification. Moreover, the five dietary-behaviour covariates were assessed using single binary clinical items without frequency or quantity thresholds rather than a validated multidimensional dietary-behaviour scale. Third, although MEDAS is a practical and validated measure of Mediterranean diet adherence, it does not capture total caloric intake, macro- and micronutrient composition, glycemic load, omega-3/omega-6 ratio, food-processing level, or the timing of dietary exposures relative to migraine attacks. Fourth, the study was conducted in a single clinical setting, which may limit generalizability. Fifth, residual confounding by socioeconomic status, education, physical activity, sleep quality, stress level, hormonal status, preventive medication use, and detailed caffeine patterns may remain. Finally, osmophobia, photophobia, and phonophobia were assessed as clinical symptoms rather than diary-based time-linked outcomes, limiting conclusions about their temporal relationship with specific dietary exposures.

Despite these limitations, this study has several strengths. It included a relatively large clinical cohort of patients diagnosed according to ICHD-3 criteria and evaluated chronic migraine status as a clinically relevant migraine phenotype. The analysis applied sequential adjustment models to assess whether the association with the Mediterranean diet persisted after accounting for demographic characteristics, BMI, disease duration, comorbidities, medication overuse, water intake, caffeine intake, sweetened carbonated beverages, and broader dietary behaviours. The use of negative binomial regression for monthly headache days also strengthened the analysis by accounting for the count-based and over-dispersed nature of headache-day data. In addition, sensitivity analyses excluding patients with medication overuse and modeling MEDAS as a continuous variable supported the robustness of the main chronic migraine finding and reduced dependence on a single categorical threshold. Future prospective studies should evaluate whether improving Mediterranean diet adherence can reduce the risk of progression from episodic to chronic migraine. Such studies should use validated dietary instruments, repeated dietary assessments, headache diaries, objective or semi-objective measures of hydration, and detailed evaluation of sleep, stress, physical activity, hormonal factors, medication use, and caffeine patterns. Randomized or structured dietary intervention studies are needed to determine whether Mediterranean diet-based interventions can modify migraine chronification risk or improve outcomes in patients with chronic migraine. Future research should also clarify whether specific Mediterranean diet components, such as olive oil, fish, legumes, vegetables, nuts, or polyphenol-rich foods, contribute more strongly to migraine-related benefits than the overall dietary pattern.

## 5. Conclusions

Higher Mediterranean diet adherence was independently associated with lower odds of chronic migraine, supporting a potential relationship between diet quality and progression to migraine chronicity. This association remained significant after adjustment for medication overuse, water intake, caffeine intake, sweetened carbonated beverage intake, and broader dietary behaviours. The association was also preserved after excluding patients with medication overuse and when MEDAS was analyzed as a continuous score, supporting the robustness of the relationship between greater Mediterranean diet adherence and lower odds of chronic migraine. The association with monthly headache days was present in clinically adjusted models but was attenuated after full dietary-behaviour adjustment. Water intake was not associated with osmophobia, caffeine intake showed only a borderline association with phonophobia, and sweetened carbonated beverage intake was not associated with migraine severity indicators. These findings support Mediterranean diet adherence as a potentially relevant lifestyle marker of migraine chronification and emphasize the need for prospective studies to determine whether targeted dietary interventions can reduce progression to chronic migraine.

## Figures and Tables

**Table 1:** Demographic and clinical characteristics according to migraine chronicity.

Variable	Episodic Migraine (*n* = 256)	Chronic Migraine (*n* = 192)	*p*-Value
Total, *n*	256	192	
Age, years	42.0 [35.0–49.0] (*n* = 256)	44.0 [37.0–53.0] (*n* = 192)	0.006
Female sex	212/256 (82.8)	161/192 (83.9)	0.770
BMI, kg/m^2^	23.8 [21.3–27.0] (*n* = 248)	23.7 [21.5–27.3] (*n* = 184)	0.549
Disease duration, years	20.0 [12.0–27.0] (*n* = 255)	21.0 [13.0–30.0] (*n* = 191)	0.077
Monthly headache days	8.0 [5.0–12.0] (*n* = 256)	20.0 [15.0–30.0] (*n* = 192)	<0.001
Severe pain days/month	7.0 [4.0–10.0] (*n* = 246)	15.0 [8.0–20.0] (*n* = 191)	<0.001
Pain intensity, VAS	8.0 [8.0–8.0] (*n* = 253)	8.0 [8.0–9.0] (*n* = 190)	0.119
Attack duration without analgesics, hours	4.0 [4.0–8.0] (*n* = 255)	4.0 [4.0–24.0] (*n* = 190)	<0.001
Medication overuse	42/256 (16.4)	110/192 (57.3)	<0.001
Photophobia	226/256 (88.3)	173/192 (90.1)	0.541
Phonophobia	202/256 (78.9)	157/192 (81.8)	0.452
Nausea	219/256 (85.5)	170/192 (88.5)	0.354
Vomiting	69/256 (27.0)	45/191 (23.6)	0.416
Osmophobia	99/255 (38.8)	70/191 (36.6)	0.640
Allodynia	12/256 (4.7)	17/192 (8.9)	0.076

Continuous variables are presented as median [IQR] with the valid *n* shown in parentheses. Categorical variables are presented as *n*/*N* (%), where *N* is the number of participants with non-missing data for that variable. Missing or invalid values were excluded. *p* values were calculated using Mann–Whitney U tests for continuous variables and chi-square or Fisher’s exact tests for categorical variables.

**Table 2:** Comorbidities and dietary/lifestyle characteristics according to migraine chronicity.

Variable	Episodic Migraine (*n* = 256)	Chronic Migraine (*n* = 192)	*p*-Value
Hypertension	20/253 (7.9)	18/191 (9.4)	0.571
Diabetes mellitus	16/253 (6.3)	15/191 (7.9)	0.531
Depression	48/253 (19.0)	45/191 (23.6)	0.240
Anxiety	46/253 (18.2)	29/191 (15.2)	0.404
Sleep apnea	4/253 (1.6)	8/191 (4.2)	0.093
MEDAS score	6.0 [4.0–8.0] (*n* = 256)	4.0 [3.0–6.0] (*n* = 192)	<0.001
At least moderate Mediterranean diet adherence (MEDAS ≥ 6)	131/256 (51.2)	54/192 (28.1)	<0.001
Daily water intake ≥1.5 L/day	131/252 (52.0)	91/186 (48.9)	0.527
Daily caffeine intake >300 mg/day	118/196 (60.2)	95/148 (64.2)	0.451
Daily sweetened carbonated beverage intake	26/224 (11.6)	18/170 (10.6)	0.750
Skipping breakfast	38/228 (16.7)	31/173 (17.9)	0.742
Eating snacks instead of proper meals	35/225 (15.6)	40/173 (23.1)	0.056
Stress-related eating	87/227 (38.3)	63/171 (36.8)	0.762
Irregular meal timing	33/226 (14.6)	30/175 (17.1)	0.488
Nocturnal eating	43/226 (19.0)	40/176 (22.7)	0.363

Continuous variables are presented as median [IQR] with the valid *n* shown in parentheses. Categorical variables are presented as *n*/*N* (%), where *N* is the number of participants with non-missing data for that variable. Missing or invalid values were excluded. *p* values were calculated using the Mann–Whitney U test for MEDAS score and chi-square or Fisher’s exact tests for categorical variables. MEDAS: Mediterranean Diet Adherence Screener.

**Table 3:** Sequential logistic regression models for the association between Mediterranean diet adherence and chronic migraine.

Model	Adjustment	N	CM Cases, *n*	Excluded, *n*	OR	95% CI	*p*-Value	AIC/BIC
Model 0	Unadjusted	448	192	0	0.373	0.251–0.556	<0.001	591.4/599.6
Model 1	Age + sex + BMI	432	184	16	0.330	0.216–0.507	<0.001	559.4/579.7
Model 2	Model 1 + disease duration	430	183	18	0.331	0.216–0.508	<0.001	558.7/583.1
Model 3	Model 2 + depression + anxiety + hypertension + diabetes + sleep apnea	429	183	19	0.338	0.220–0.520	<0.001	564.8/609.5
Model 4	Model 3 + medication overuse	429	183	19	0.346	0.216–0.554	<0.001	498.7/547.5
Model 5	Model 4 + daily water intake	422	178	26	0.306	0.188–0.499	<0.001	491.1/543.6
Model 6	Model 5 + daily caffeine intake	325	138	123	0.301	0.172–0.526	<0.001	386.3/439.2
Model 7	Model 6 + sweetened carbonated beverage intake + dietary habit variables	283	122	165	0.360	0.193–0.674	0.001	339.7/412.6

Outcome: chronic migraine status. Main exposure: at least moderate Mediterranean diet adherence (MEDAS ≥ 6 vs. 0–5). CM cases indicate the number of participants with chronic migraine in each model-specific analytic sample. Excluded indicates the number excluded from the original sample of 448 because of missing model variables. OR < 1 indicates lower odds of chronic migraine. AIC, Akaike information criterion; BIC, Bayesian information criterion. Because analytic sample sizes differ across models, AIC and BIC should not be compared directly between all sequential models.

**Table 4:** Sequential negative binomial regression models for the association between Mediterranean diet adherence and monthly headache days.

Model	Adjustment	*N*	CM Cases, *n*	Excluded, *n*	IRR	95% CI	*p*-Value	AIC/BIC
Model 0	Unadjusted	448	192	0	0.814	0.720–0.920	0.001	3154.4/3166.7
Model 1	Age + sex + BMI	432	184	16	0.777	0.686–0.880	<0.001	3026.0/3050.4
Model 2	Model 1 + disease duration	430	183	18	0.778	0.687–0.882	<0.001	3012.9/3041.4
Model 3	Model 2 + depression + anxiety + hypertension + diabetes + sleep apnea	429	183	19	0.788	0.696–0.892	<0.001	3010.4/3059.2
Model 4	Model 3 + medication overuse	429	183	19	0.825	0.733–0.928	0.001	2962.3/3015.1
Model 5	Model 4 + daily water intake	422	178	26	0.817	0.723–0.923	0.001	2916.5/2973.2
Model 6	Model 5 + daily caffeine intake	325	138	123	0.855	0.743–0.985	0.030	2258.8/2315.6
Model 7	Model 6 + sweetened carbonated beverage intake + dietary habit variables	283	122	165	0.883	0.755–1.033	0.120	1979.6/2056.2

Outcome: monthly headache days. Main exposure: at least moderate Mediterranean diet adherence (MEDAS ≥ 6 vs. 0–5). CM cases are reported descriptively for each model-specific analytic sample; the modeled outcome is monthly headache-day count. Excluded indicates the number excluded from the original sample of 448 because of missing model variables. IRR < 1 indicates fewer monthly headache days. AIC, Akaike information criterion; BIC, Bayesian information criterion. Because analytic sample sizes differ across models, AIC and BIC should not be compared directly between all sequential models.

**Table 5:** Sensitivity analyses excluding patients with medication overuse.

Outcome	Model Specification	*N*	Measure	Effect Estimate	95% CI	*p*-Value
Chronic migraine	Model 1	287	OR	0.377	0.212–0.669	<0.001
Chronic migraine	Clinically adjusted	286	OR	0.399	0.222–0.715	0.002
Chronic migraine	Full lifestyle	191	OR	0.406	0.186–0.889	0.024
Monthly headache days	Model 1	287	IRR	0.809	0.687–0.954	0.011
Monthly headache days	Clinically adjusted	286	IRR	0.824	0.699–0.970	0.020
Monthly headache days	Full lifestyle	191	IRR	0.891	0.723–1.098	0.278

Analyses were restricted to participants without medication overuse. The exposure was at least moderate Mediterranean diet adherence (MEDAS ≥ 6 vs. 0–5). Model specifications followed the prespecified sequential adjustment framework described in Section 2.5. CM, chronic migraine; OR, odds ratio; IRR, incidence rate ratio.

**Table 6:** Exploratory analyses of water, caffeine, and sweetened carbonated beverage intake.

Exposure	Outcome/Model Specification	*N*	Measure	Effect Estimate	95% CI	*p*-Value
Water intake ≥1.5 L/day	Osmophobia; adjusted for monthly headache days	436	OR	1.320	0.884–1.972	0.175
Water intake ≥1.5 L/day	Osmophobia; adjusted for chronic migraine status	436	OR	1.301	0.870–1.946	0.201
Caffeine intake >300 mg/day	Phonophobia; adjusted for monthly headache days	344	OR	1.733	0.986–3.047	0.056
Caffeine intake >300 mg/day	Phonophobia; adjusted for chronic migraine status	344	OR	1.727	0.983–3.036	0.058
Caffeine intake >300 mg/day	Photophobia; adjusted for monthly headache days	344	OR	1.406	0.664–2.978	0.374
Caffeine intake >300 mg/day	Photophobia; adjusted for chronic migraine status	344	OR	1.394	0.658–2.952	0.385
Daily sweetened carbonated beverage intake	Chronic migraine	382	OR	0.903	0.436–1.871	0.784
Daily sweetened carbonated beverage intake	Monthly headache days	382	IRR	1.087	0.898–1.315	0.390
Daily sweetened carbonated beverage intake	Pain intensity	377	β	−0.001	−0.305–0.302	0.993
Daily sweetened carbonated beverage intake	Medication overuse	382	OR	0.874	0.408–1.873	0.730

Water- and caffeine-exposure models were adjusted for age, sex, medication overuse, Mediterranean diet adherence, and either monthly headache days or chronic migraine status, as indicated. Sweetened carbonated beverage models were adjusted for age, sex, BMI, Mediterranean diet adherence, and medication overuse, where appropriate. These analyses were exploratory. OR, odds ratio; IRR, incidence rate ratio; β, regression coefficient.

## Data Availability

The data presented in this study are available on request from the corresponding author due to privacy or ethical restrictions.

## Supplementary Materials

The following supporting information can be downloaded at: https://www.mdpi.com/article/10.3390/nu18152563/s1, Table S1: Sensitivity Analyses of the Associations Between MEDAS Score, Modeled as a Continuous Variable, and Chronic Migraine and Monthly Headache Days.
