# Supplementary material for: Greater Mediterranean Diet Adherence Is Associated with Lower Odds of Chronic Migraine: A Retrospective Observational Study
Source: Nutrients. 2026 Aug 5;18(15):2563. doi: 10.3390/nu18152563 (PMC13468569; doi:10.3390/nu18152563)
Supplement: Supplementary file 1 [file nutrients-18-02563-s001.zip › nutrients-4415666-supplementary.pdf]

Supplementary Table S1. Sensitivity Analyses of the Associations Between MEDAS Score, Modeled as a Continuous Variable, and Chronic Migraine and Monthly Headache Days

Outcome	Model	N	Effect	Estimate	95% CI	p
Chronic migraine	Model 0	448	OR	0.89	0.831–0.954	<0.001
Chronic migraine	Model 1	432	OR	0.882	0.821–0.947	<0.001
Chronic migraine	Model 2	430	OR	0.881	0.820–0.946	<0.001
Chronic migraine	Model 3	429	OR	0.883	0.822–0.949	<0.001
Chronic migraine	Model 4	429	OR	0.862	0.796–0.933	<0.001
Chronic migraine	Model 5	422	OR	0.847	0.781–0.919	<0.001
Chronic migraine	Model 6	325	OR	0.836	0.761–0.917	<0.001
Chronic migraine	Model 7	283	OR	0.842	0.758–0.936	0.001
Monthly headache days	Model 0	448	IRR	0.984	0.963–1.005	0.142
Monthly headache days	Model 1	432	IRR	0.979	0.958–1.001	0.058
Monthly headache days	Model 2	430	IRR	0.979	0.958–1.000	0.054
Monthly headache days	Model 3	429	IRR	0.98	0.959–1.002	0.075
Monthly headache days	Model 4	429	IRR	0.98	0.960–1.000	0.051
Monthly headache days	Model 5	422	IRR	0.978	0.958–0.999	0.043

Monthly headache days	Model 6	325	IRR	0.982	0.959–1.007	0.152
Monthly headache days	Model 7	283	IRR	0.989	0.963–1.016	0.433

Effect estimates are reported per one-point increase in MEDAS score. OR: odds ratio; IRR: incidence rate ratio; CI: confidence interval; MEDAS: Mediterranean Diet Adherence Screener.
